# Assessment of General Public’s Knowledge and Opinions towards Antibiotic Use and Bacterial Resistance: A Cross-Sectional Study in an Urban Setting, Rufisque, Senegal

**DOI:** 10.3390/pharmacy6040103

**Published:** 2018-09-20

**Authors:** Oumar Bassoum, Ndèye Marème Sougou, Mayassine Diongue, Mamadou Makhtar Mbacke Lèye, Mouhamad Mbodji, Djibril Fall, Ibrahima Seck, Adama Faye, Anta Tal-Dia

**Affiliations:** 1 Department of Public Health and Preventive Medicine, Faculty of Medicine, Pharmacy and Odontology, University Cheikh Anta Diop, 5005 Dakar-Fann, Senegal; ndeyemareme.sougou@ucad.edu.sn (N.M.S.); mayassine.diongue@ucad.edu.sn (M.D.); mamadou.leye@ucad.edu.sn (M.M.M.L.); ibrahima.seck@ucad.edu.sn (I.S.); adama.faye@ucad.edu.sn (A.F.); anta.dia@ucad.edu.sn (A.T.-D.); 2 Institute of Health and Development, University Cheikh Anta Diop, 16390 Dakar-Fann, Senegal; 3 Laboratory of Therapeutic and Organic Chemistry, Faculty of Medicine, Pharmacy and Odontology, University Cheikh Anta Diop, 5005 Dakar-Fann, Senegal; mbodjimouha28@gmail.com (M.M.); djibril.fall@ucad.edu.sn (D.F.)

**Keywords:** knowledge, opinions, general public, antibiotic use, bacterial resistance, Rufisque, Senegal

## Abstract

*Background:* Bacterial resistance is a major public health problem worldwide. One solution to this scourge is to sensitize the general public on rational use of antibiotics. Our goal was to assess people’s knowledge and opinions about antibiotic use and bacterial resistance in an urban setting. *Method:* We performed a cross-sectional study. A convenience sampling was done. A questionnaire was administered to 400 persons during face-to-face interviews. *Results:* Most respondents thought that antibiotics are effective against colds/flu (69.8%), cough (72.3%) and sore throat (64.4%). At the same time, 42.8% stated that antibiotic therapy can be stopped as soon as the symptoms disappear. Only 8.8% and 41.8% of people knew that handwashing and vaccination prevented bacterial resistance. Globally, 7% of people had a good knowledge. Socio-demographic variables were not associated with the level of knowledge. The main sources of information were entourage and pharmacy staff. Regarding the opinions, 78.3% of surveyed participants the people thought that that people overuse antibiotics. Additionally, 28% said that they have no role to play against bacterial resistance. *Conclusion:* People living in an urban setting had a low knowledge about antibiotic use and bacterial resistance. There is a need to implement awareness campaigns. Further studies on population practices toward antibiotic use are necessary.

## 1. Introduction

Antibiotics are a category of antimicrobial drugs that revolutionized modern medicine [1]. They have improved health and saved millions of lives [2,3]. Thus, they were considered as “miracle drugs” into thinking that infectious diseases would become a problem of the past [3]. However, this hope has not been realized to the extent that their effectiveness is compromised by the emergence of bacterial resistance [4]. The World Health Organization (WHO), in his first global report on surveillance of antimicrobial resistance, estimates that it is occurring across many infectious agents and has spread to all regions of the world [5]. This report reveals high levels of resistance to last resort treatment for life-threatening infections caused by *Klebsiella pneumoniae*. In addition, *Escherichia coli* has become resistant to fluoroquinolones, which are among the most widely used antibiotics for the treatment of urinary tract infections. In 2014, the total number of multidrug-resistant tuberculosis cases is estimated at 480,000, of which 10% were extensively drug-resistant [6]. In developing countries, Antimicrobial resistance is a significant issue for health care. In Australia, vancomycin-resistant enterococci and community-acquired methicillin-resistant Staphylococcus aureus have emerged as major antibiotic resistant bacteria [7]. In United States (US), the Centers for Disease Control and Prevention (CDC) have published a list of antibiotic resistant bacteria. The threat level of each bacteria is categorized as urgent (e.g., carbapenem-resistant Enterobacteriaceae), serious (e.g., drug-resistant *Salmonella Typhi*) and concerning (e.g., Erythromycin-resistant Group A streptococcus) [8].

In sub-Saharan Africa, the extent of the problem is not sufficiently known due to the unavailability of data [5], but recent studies have shown resistance to the most commonly used antibiotics such as amoxicillin, ampicillin, gentamicin and ceftriaxone [9,10,11]. In Senegal, studies have found high levels of resistance to tetracycline, cotrimoxazole, ampicillin, quinolones [12] and third-generation cephalosporins [13].

Bacterial resistance is a natural phenomenon [14]. However, it can be accelerated by the overuse of antibiotics in both human and veterinary medicine [1,15,16,17]. About 80% of antibiotics are used in community settings [18,19]. In developing countries, people can obtain them not only on prescription by exerting pressure on health workers but also on self-medication [1,18,19,20,21]. Low adherence to treatment is also recognized as a risk factor for bacterial resistance [3]. According to the WHO, many patients do not complete the prescribed treatment [17]. Misinformation, saving antibiotic for later, response to advertising are other factors contributing to bacterial resistance [22].

The latter is considered to be one of the greatest threats to human health [23]. Antimicrobial resistance, in particular antibiotic resistance, causes 700,000 deaths per year [1]. In the US, more than two million people are sickened every year with antibiotic-resistant infections. Of these, about 23,000 deaths are recorded every year. The loss of effective antibiotic treatments will compromise the treatment of most common bacterial diseases. It will also undermine the management of infectious complications in people with other diseases. For example, certain medical procedure such as joint replacements, organ transplants, cancer therapy could again become much more dangerous. In addition, chronic diseases such as diabetes, asthma would very difficult to treat [8]. Otherwise, according to the World Bank, antibiotic-resistant infections could hinder the achievement of sustainable development goals [24] and push 28 million people into poverty in 2050 [25].

Recognizing this alarming situation, WHO has developed a global action plan to prevent and control antimicrobial resistance [26]. This plan is structured around fives strategic objectives: (i) to improve awareness and understanding of antimicrobial resistance through effective communication, education and training; (ii) to strengthen the knowledge and evidence base through surveillance and research; (iii) to reduce the incidence of infection through effective sanitation, hygiene and infection prevention measures; (iv) to optimize the use of antimicrobial medicines in human and animal health; (v) to develop the economic case for sustainable investment that takes account of the needs of all countries and to increase investment in new medicines, diagnostic tools, vaccines and other interventions. Achieving these objectives requires the involvement of several stakeholders. Thus, the United Nations Food and Agriculture Program and the World Organization for Animal Health support this plan as part of ‘One Health’ approach to promote the rational use of antibiotics in human and animal health, agriculture and food chain [26]. According to this plan, understanding social science and behavior is also important for the achievement of objectives 1, 2, 3 and 4 [26].

In Senegal, a framework for fighting against bacterial resistance exists [27]. A multi-sectoral action plan against antimicrobial resistance is being finalized [28]. The country’s ability to cope with bacterial resistance has been recently evaluated [29]. One of the main recommendations is the sensitization of the populations about antibiotics use [29]. However, understanding the knowledge level of populations about antibiotic use and bacterial resistance could better implement awareness campaigns. Thus, studies are conducted in several countries [30,31,32]. Ours fits in this dynamic. These studies found several misunderstanding about antibiotic use. For example, the respondents thought that they should stop taking antibiotics when they feel better [30]. They did not believe that they contribute to the development of bacterial resistance [31] and did not know that misuse of antibiotics can lead to bacterial resistance [32]. To the best of our knowledge, our study is the first in Senegal. It enriches global research on the general public’s awareness about bacterial resistance. In addition, it will allow health authorities to make decisions and target awareness messages. Our goal was to evaluate the knowledge and opinions of the general public about antibiotic use and bacterial resistance.

## 2. Materials and Methods

### 2.1. Study Setting

The study took place in the Rufisque bus station. We selected this site for easy access to general public. It also saves cost and time. Rufisque is the departmental county town of the same name and belongs to the region of Dakar. It is subdivided into three district municipalities. In 2017, its population was estimated at 248.696 inhabitants [33]. City data are limited, but in the department, the average age of the population is 24 years old. Rates of education, literacy and unemployment are estimated at 75%, 45% and 15.5%, respectively. The average household size is 8.3 people. Promiscuity is a concern in the department since 15.7% of households have three people per residential room [34]. About 52% of households have a faucet in their dwelling, while 17.8% use the public tap or the standpipe [34]. In the department, only 19.7% of households have a specific place for washing hands with soap [35]. Sanitation is very little developed [36].

### 2.2. Type and Period of Study

We conducted a cross-sectional study of the general public’s knowledge and opinions towards antibiotic use and bacterial resistance. The data collection took place from 25 November to 25 December 2017.

### 2.3. Study Population

The study population consisted of persons attending the Rufisque bus station. This included anyone over the age of 18 years, who had agreed to participate in the study and who was able to name one antibiotic. Health professionals such as doctors, nurses, midwives and pharmacists were excluded from the study.

### 2.4. Sample Size

We used the Raosoft sample size calculator [37] to estimate the sample size. The hypotheses were the following:

(i) response distribution: 50%;
(ii) source population size: 20,000;
(iii) desired level of confidence: 95%;
(iv) margin of error: 5%.

In Senegal, there are no previously studies that assessed population’s knowledge about antibiotic use and bacterial resistance. Therefore, we did not know the response distribution. We assumed that the response distribution for each question would be 50% using a margin of error of 5% and a confidence level of 95%. These hypotheses maximize the sample size [37]. In addition, the source population size (people attending the bus station) was not known. According to Raosoft sample size calculator, we had set it at 20,000 [37]. Thus, the sample size was 377. We rounded the size to 400 participants in order to optimize its representativeness.

### 2.5. Sampling Procedure

For reasons of technical and financial feasibility, we performed a non-probability sampling (convenience sampling). Participants were recruited among the travelers encountered in front of the Rufisque bus station. Each person who rejected to participate in the study was replaced by another until the required number of subjects was reached. The participation was voluntary.

### 2.6. Data Collection

#### 2.6.1. Data Collection Instrument

Our data collection instrument was a questionnaire which was developed by two co-authors. We did not perform the test-retest of the questionnaire. However, it was reviewed and improved by all co-authors, some of whom have an extensive experience in the area of public health, epidemiology and medicines. They have made relevant comments in both content and form. Some of the questions were reformulated while others were deleted. The questionnaire was also previously tested on 16 people. Then, we held a meeting with all the co-authors. Corrections were made based on feedbacks from the pre-test. The final version included a filter question to identify participant who are able to name one antibiotic. It consisted of four sections. The first was related to socio-demographic characteristics and included seven items. The second section assessed the level of knowledge through twelve questions while the sources of information were collected in the third section. Reponses on knowledge had three options: ‘true’, ‘false’ or ‘don’t know’. The correct option is ‘false’ for Q1–Q7 and ‘true’ for Q8–Q12. The incorrect option is ‘true’ or ‘don’t know’ for Q 1–Q7 and ‘false’ or ‘don’t know’ for Q8–Q12. The level of knowledge was evaluated as follows: One point was given for each correct answer and zero for each incorrect answer.

The overall score on knowledge was obtained by adding up the scores of the answers. The minimum and maximum scores were 0 and 12 points, respectively. The level of knowledge was classified according to the method described by Essi MJ and Njoya O [38]:

- “less than 25% of correct answers” = bad
- “more than or equal to 25% of correct answers and less than 50% of correct answers” = insufficient
- “more than or equal to 50% of correct answers and less than 70% of correct answers” = average
- “more than or equal to 70% of correct answers” = good

Finally, opinions towards antibiotic use and bacterial resistance were collected in the fourth section. This section consisted of four questions and the response options were ‘yes’, ‘no’ or ‘no opinion’.

#### 2.6.2. Data Collection Method

The data collection method used was a face-to-face interview conducted by one interviewer. The latter stood in front of the Rufisque bus station and questioned travelers who met the inclusion criteria. The interviewer was a sixth-year pharmacy student at the Cheikh Anta Diop University in Dakar, Senegal. He was trained within two days. The training consisted of sensitizing him about the context, rationale and objectives of the study. We presented him the data collection method and instrument. He was also trained in questionnaire administration before and after the pre-test.

#### 2.6.3. Data Collected

The data collected were socio-demographic characteristics: age (18–30, 31–40, 41–50, 51–60, >60 years), sex (male/female), marital status (married/unmarried), professional status (employed/unemployed), education (yes/no), distance between place of residence and a health facility (≤300 m/>300 m) and distance between place of residence and a community pharmacy (≤300 m/>300 m).As for knowledge, the data were related to antibiotic use in colds/flu, sore throat, cough, diarrhea, fever and fatigue. Knowledge about risk factors for bacterial resistance, strategies to prevent bacterial resistance (hand washing, vaccination) and impact of antibiotic use in animals on human health was also collected. Finally, the participants’ opinions on the levels of antibiotic consumption in the community, sensitization during consultation in health facilities or dispensing of medicines in pharmacies and their role in the fight against bacterial resistance were collected.

### 2.7. Data Analysis

The data was entered into Microsoft Excel 2010. The statistical analysis consisted of two parts. The first described the distribution of the variables. The qualitative variables were expressed as frequency and percentage. Knowledge score is expressed as mean ± standard deviation. The second part was analytical. It studied the relationship between knowledge and sociodemographic variables. For this purpose, we performed a bivariate analysis (χ^2^ test, OR with 95% confidence interval) and logistic regression. According to Hosmer DW and Lemeshow S, variables with *p*-value less than 0.25 in bivariate analysis were included in the logistic regression [39]. These authors show that use *p*-value of 0.05 as inclusion criteria for logistic regression model often fails to identify variables known to be important [39]. The significance level was 0.05.The analyzes were carried out using Epi Info 7.

### 2.8. Ethical Considerations

Our research involved human subjects. However, it was an observational study which was carried out as part of a Pharm. D thesis. We conducted this study in conditions that did not expose the participants to physical risks or discomfort (example: there was no blood test). It did not harm the environment either. The administration of the questionnaire lasted approximately 10 min. Therefore, it did not irritate the participant.

All subjects gave their informed consent for inclusion before they participated in the study and after explaining the context, rationale and objectives of the study. Participation was voluntary. Each participant was informed that he or she had the opportunity to withdraw from the study at any time without being compelled to justify oneself. The data were collected anonymously and confidentially.

The Cheikh Anta Diop University Research Ethics Board approved this study.

## 3. Results

A total of 400 participants were included in our study. The questionnaire was administered to all 400 people (100%) who agreed to participate in the survey. Only 22 persons rejected to participate because they were in a hurry. These persons were replaced by others who were readily available to the interviewer. All forms were completely filled (100%).

### 3.1. Socio-Demographic Characteristics

The 31–40 age group was the most represented (32.5%). It was followed by 18–30 age group (28.5%). People over the age of 60 accounted for 6.5% (Table 1).More than half of the participants were male (55%) and married (55.5%) (Table 1). Sixty-two percent (62%) and nearly half of all participants (48.75%) were employed and educated, respectively (Table 1).In addition, 49.25% and 68.5% lived in close proximity to a health facility and a community pharmacy, respectively (Table 1).

### 3.2. Filter Question

**Figure 1:**
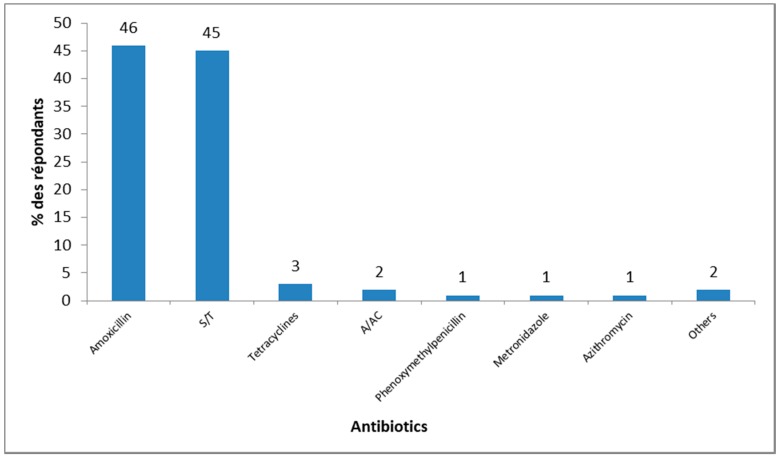
Antibiotic cited by participants, Rufisque, Senegal, 2017 (N = 400). S/T = Sulfamethoxazole/Trimethoprim, A/AC = Amoxicillin/clavulanic acid, Others = Doxycycline, Gentamicin, Penicillins, Oxacillin.

All people included in the study cited one antibiotic. Among them, most mentioned amoxicillin (46%) and sulfamethoxazole/trimethoprim (45%) (Figure 1). Amoxicillin/clavulanic acid was indicated by 2% of respondents (Figure 1).

### 3.3. Knowledge

Most respondents mistakenly thought that antibiotics are effective against colds/flu (69.8%), cough (72.3%), and sore throat (64.4%) (Table 2).At the same time, 42.8% of surveyed participants said that one should stop taking an antibiotic treatment as soon as one feels better, while 83.8% knew that high antibiotic consumption can lead to bacterial resistance (Table 2).A quarter (25.5%) of people was aware that poor patient compliance with antibiotic treatment can be harmful for people (Table 2). In addition, only 8.8% and 41.8% agreed that hand washing and vaccination can prevent bacterial resistance (Table 2).As for the excessive use of antibiotics in animals, 67.5% of the respondents thought that it has a negative impact on human health (Table 2).

### 3.4. Knowledge Level Classification

The average score was 4.5 ± 1.96. The highest score was 10 while the lowest was 0. The mode was 4. The median, first quartile and third quartile were 4, 3 and 6, respectively. The level of knowledge was bad and insufficient in 58 (14.5%) and 218 (54.5%) participants, respectively. In contrast, only 96 (24%) and 28 (7%) had average and good level of knowledge, respectively. There were no significant associations between the respondents’ socio-demographic characteristics and level of knowledge (Table 3).

### 3.5. Sources of Knowledge

**Figure 2:**
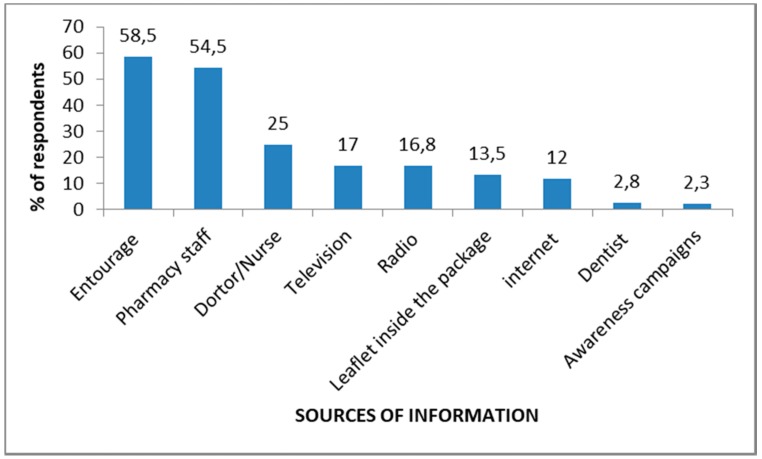
Sources of information, Rufisque, Senegal, 2017 (N = 400).

The top three sources of information cited by the respondents were entourage (family or friends) (58.5), pharmacy staff (54.5%), doctor/nurse (25%) (Figure 2). Television and radio were reported in 17% and 16.8%, respectively (Figure 2).

### 3.6. Opinions

Nearly eight out of ten people (78.3%) thought that the population overuses antibiotics (Table 4). At the same time, 28% and 53.5% felt that they were not getting enough information about the consequences of poor adherence to antibiotic treatment, during consultations and dispensing, respectively (Table 4). About 45% of respondents thought that they do not have a large role to play in the fight against bacterial resistance (Table 4).

## 4. Discussion

We aimed to assess the general public’s knowledge and opinions about antibiotic use and bacterial resistance in an urban setting.

Our study showed that the two most cited antibiotics were amoxicillin (46%) and sulfamethoxazole/trimethoprim (45%). In a study conducted in Kenya, they were also the most mentioned by participants [40]. These results assume that the population is accustomed to using these two broad-spectrum antibiotics. They are among the most accessible antibiotics in Africa [40]. Studies conducted in Senegal and Angola seem to confirm our hypothesis since amoxicillin and sulfamethoxazole/trimethoprim were among the most common antibiotics found in households [41,42]. The use of broad-spectrum antibiotics can lead to an intestinal flora imbalance and the spread of bacterial resistance [43]. In Senegal, cases of bacterial resistance against sulfamethoxazole/trimethoprim were reported in patients with community-acquired urinary tract infection [44]. So these antibiotics should be used carefully. Further studies should be conducted in order to understand the reasons underlying the familiarity of the population with these two antibiotics.

This study has shown many misconceptions about antibiotic use. Indeed, more than three out of five people wrongly thought that antibiotics work against cough (72.3%), colds/flu (69.8%), sore throat (64.3%) and diarrhea (35.3%). Similar results were found in a WHO study conducted in several countries [30]. According to this study, 64% and 70% of respondents thought that antibiotics have an effect against colds/flu and sore throat [30], respectively. In Saudi Arabia, 66.4% of the population thought that antibiotics are effective against colds or coughs [45]. In most cases, these infections are of viral origin [46,47,48,49]. Antibiotics are not routinely recommended as they cannot kill viruses [46,47,48]. These misconceptions may be due to the propensity of health professionals to prescribe or dispense antibiotics against these diseases witch are self-limiting [45,50]. According to Pelluchi et al., prescribers use antibiotics to prevent complications, relieve symptoms or satisfy patient’s desires [46]. For example, in the United States, nearly 68% of patients with respiratory infections are unnecessarily treated with antibiotics [1]. The misunderstanding about antibiotic use clearly demonstrates the need to sensitize populations on the proven efficacy of analgesics against acute respiratory infections [46]. This awareness is fundamental because the unnecessary antibiotic use exposes them to side effects such as diarrhea, vomiting and rash, bacterial flora imbalance and bacterial resistance [46]. Additionally, continuing education for health professionals on rational use of antibiotics would be necessary. This training should be an opportunity to encourage them not only to resist against patient pressure but also to prescribe laboratory diagnosis in order to identify the germ.

In our study, 42.8% of respondents incorrectly supported the idea that antibiotic treatment should be discontinued as soon as symptoms disappear. This proportion was comparable to those found in Angola (40%) [51] and Malaysia (41.9%) [52], but lower than those found in Kenya (83.6%; 93.4%) [40] and India (65.55%) [53]. The lack of knowledge about antibiotic use could concretely result in the arbitrary cessation of treatment. For example, in the United Kingdom and Ethiopia, patients admitted to having stopped treatment as soon as they felt better [3,54]. This inadequate practice exposes them to the risk of recurrence and bacterial resistance [53].

Overuse of antibiotics is a risk factor for bacterial resistance. This relationship is widely mentioned in the literature [3,55,56]. In our study, almost five out of six people (83.8%) recognized it. This proportion was comparable to that found in Romania (87%) [57], while it was higher than those found in Malaysia (59.1%) [58], in Egypt (60.7%) [59], in Namibia (72%) [60], and in a systematic review (70%) [32]. On the one hand, overuse of antibiotics could result from inappropriate prescriptions which are very common in sub-Saharan Africa [3,61]. On the other hand, it could be interpreted as a consequence of self-medication which itself is exacerbated by the sale of drugs, including antibiotics, ‘over-the-counter’ [1,3,62]. In West Africa, antibiotics can even be obtained in popular markets [63]. Solutions to reduce unnecessary use of antibiotic exist. It includes public education [3], on-going in-service education for prescribers especially those with a short experience [64] and regulation of the pharmaceutical sector [65].

In our study, few people (25.5%) knew that poor patient compliance with antibiotic treatment may be harmful to people. Indeed, it can lead to resistant strains which can spread across borders [66]. The transmission is favored by poverty, which is a characteristic of developing countries [66]. In fact, poor people have limited access to care services [66]. Thus, they represent sources of transmission when they suffer from communicable diseases [66]. Considering poor patient compliance as a personal problem demonstrates clearly that the general public is not well aware of the risk factors and extent of bacterial resistance.

Nearly 68% of respondents agreed that the inappropriate use of antibiotics in animals can have a negative impact on human health. Most antibiotics used in animals belong to the same classes of antimicrobials used in human medicine [17,67]. In some African countries, including Senegal, more than 80% of animal products contain drug residues [68]. The use of these antibiotics for the promotion of growth, generally at low doses, is a risk factor for bacterial resistance [17]. The resistant strains found in animal productions can be transmitted to humans through the food chain and compromise the efficacy of antimicrobials in human medicine [69]. Therefore, the consideration of agricultural and livestock sectors in the prevention and control of bacterial resistance is essential. The ‘one health’ approach is a great opportunity to promote the rational use of antibiotics in humans and animals.

The results of our study revealed that only 8.8% and 41.8% of surveyed participants recognized that regular hand washing with soap and vaccination can prevent bacterial resistance, respectively. However, these two strategies avoid contracting infectious diseases. So they reduce the need for antibiotics. For example, several studies have shown that the pneumococcal vaccine has reduced antibiotic resistance in both children and adults [70,71]. As for the hand washing, it is admitted that it diminish the occurrence of diarrheal and respiratory diseases [72]. These results show that the fight against bacterial resistance necessarily involves sensitizing the populations in order to make them understand the importance of these two high-impact strategies. However, while hand washing is an easily achievable practice, the major challenge for Africa remains the availability and accessibility of vaccines.

Overall, almost three people (69%) out of four had bad or insufficient level of knowledge toward antibiotic use and bacterial resistance. This situation is comparable to that found in Kuwait (47%) [73], Lithuania (61.1%) [74] and Egypt (59.6%) [59]. In addition, our study did not reveal a significant relationship between the good level of knowledge and socio-demographic characteristics. However, other studies have shown the influence of sex, age [75] and level of education [74,75] on knowledge. In our study, the non-significant association between socio-demographic characteristics and the good level of knowledge could be explained by the fact that we used a non-probability sampling technique. Further studies are needed in order to identify the socio-demographic determinants of the knowledge regarding antibiotic use and bacterial resistance.

The main sources of information identified in this study were the entourage (58.5%), the pharmacy staff (54.5%) and the Doctors/nurses (25%). These three sources of information were mostly identified in Kenya [40] and Lithuania [74]. In contrast, awareness campaigns were reported by a small proportion of respondents (2.3%). Our study showed that health authorities should set up and intensify training and awareness programs for health professionals so that they can enhance people’s knowledge through reliable information. Moreover, about 17%, 16.8% and 12% of respondents cited television, radio and internet as sources of information, respectively. These results showed that information on antibiotic use is not widely disseminated in these communication media despite their strong presence in households and among young people. However, the effectiveness of awareness campaigns disseminated through television is proven in several countries [31].

Most (78.3%) of surveyed participants believed that the population overuses antibiotics. A recent study corroborates this view. It shows that global consumption of antibiotics increased by 65% between 2000 and 2015 [76]. This increase is stronger in developing countries [76]. Awareness of our study population about high rate of antibiotic consumption is an opportunity to encourage the community to use antibiotics only when they are needed. This would allow us to preserve the effectiveness of the current therapeutic arsenal. Hence, the post-antibiotic era so feared could not come to pass.

Paradoxically, 28% of the participants believed that they do not have a great role to play in fighting bacterial resistance. A similar opinion was found in a recent study in which adolescents thought that solving the problem of bacterial resistance is the responsibility of prescribers [77]. The WHO survey found that 57% of respondents though they cannot do much to stop bacterial resistance [30]. This shows that the population has not been aware of the actions they could initiate against bacterial resistance. WHO has identified five relevant actions [78]:

- ‘Only use antibiotics when prescribed by a certified health professional’
- ‘Always take the full prescription, even if you feel better’
- ‘Never use left-over antibiotics’
- ‘Never share antibiotics with others’
- ‘Prevent infections by regularly washing your hands, avoiding close contact with sick people and keeping your vaccinations up to date’

But, it would be necessary to multiply the awareness campaigns for a strict application of these five recommendations.

Furthermore, communication between patients and healthcare professionals is essential to combat bacterial resistance. In this study, only 28% of people thought that they did not receive enough information about bacterial resistance during consultation in health facilities. This could be due to a lack of knowledge among prescribers who, in some countries such as Ghana, face limited access to the internet, lack of information on the patterns of resistance [79]. In addition, 45.8% of participants felt that they receive enough information from pharmacy staff. This proves that pharmacists have a very important role in the fight against bacterial resistance given their proximity to populations. However, to assume this role, the pharmacist needs to update his knowledge in accordance with the ‘Seven-Star Pharmacist concept’ [80]. To this end, the health authorities should collaborate with pharmacists in order to set up a continuing education program on bacterial resistance.

Our study has two limitations. First, the sampling is non-probabilistic. According to Bornstein MH et al., in this type of sampling, participants were selected because they are easily accessible [81]. In this study, the sample consisted of individuals attending the Rufisque bus station. So, there was a low chance to include some persons such as those who have their own car. Therefore, the sample is not representative of the entire population. Second, the survey was limited to an urban area. In general, socio-economic status may be different between urban and rural areas. Consequently, people’s knowledge and perceptions towards antibiotic use and bacterial resistance may vary according to area of residence. In the light of these considerations, our results would not generalizable. However, to the best of our knowledge, this is the first study in Senegal. Thus, it is a starting point for further research that focuses both on urban and rural settings using a probability sampling.

## 5. Conclusions

Our study showed a weak knowledge and misconceptions about antibiotic use and bacterial resistance among people living in an urban setting. Therefore, sensitizing the general public is necessary. Health professionals should regularly undergo continuing education sessions so that they can provide reliable information to patients. Conducting further studies on practices and attitudes towards antibiotic use would help to better target the awareness campaigns and training.

## Figures and Tables

**Table 1:** Socio-demographic characteristics’ participants, Rufisque, Senegal, 2017 (N = 400).

Socio-Demographic Characteristics	n	%
Age (years)		
[18–30]	114	28.5
[31–40]	130	32.5
[41–50]	87	21.8
[51–60]	43	10.8
>60	26	6.5
Sex		
Male	220	55
Female	180	45
Marital status		
Married	222	55.5
Unmarried	178	44.5
Professional status		
Employed	248	62
Unemployed	152	38
Education		
Yes	195	48.75
No	205	51.25
Close proximity to a health facility		
Yes (<300 m)	197	49.25
No (>300 m)	203	50.75
Close proximity to a community pharmacy		
Yes (<300 m)	274	68.5
No (>300 m)	126	31.5

**Table 2:** Participants’ knowledge towards antibiotic use and bacterial resistance, Rufisque, Senegal, 2017 (N = 400).

Statements	True n (%)	False n (%)	Don’t Know n (%)
Antibiotics work against:			
1. Cold/Flu	279 (69.8)	97 (24.3)	24 (6.0)
2. Cough	289 (72.3)	85 (21.3)	26 (6.5)
3. Sore throat	257 (64.3)	84 (21.0)	59 (14.8)
4. Diarrhea	141 (35.3)	184 (46.0)	75 (18.8)
5. Fever	167 (41.8)	205 (51.3)	28 (7.0)
6. Fatigue	203 (50.8)	153 (38.3)	44 (11.0)
7. One should stop taking an antibiotic treatment as soon as one feels better	171 (42.8)	227 (56.8)	2 (0.5)
8. High antibiotic consumption can lead to bacterial resistance	335 (83.8)	54(13.5)	11(2.8)
9. Poor patient compliance with antibiotic treatment may be harmful to people	102 (25.5)	267 (66.8)	31 (7.8)
10. Handwashing can prevent bacterial resistance	35 (8.8)	228 (57)	137 (34.3)
11. Vaccination can prevent bacterial resistance	167 (41.8)	205 (51.3)	28 (7)
12. Inappropriate antibiotic use in animals can result in negative impact on human health	270 (67.5)	117 (29.3)	13 (3.3)

Expected responses: False (Q1–Q7) and True (Q8–Q12).

**Table 3:** Bivariate and multivariate analysis of the relationship between good level of knowledge and socio-demographic characteristics, Rufisque, Senegal, 2017 (N = 400).

SDC	GLN		BA			MA		
	Yes	No	OR	IC 95%	*p*-Value	OR	IC 95%	*p*-Value
Age								
≤40	22	222	2.4	[1.0–6.2]	0.048	1.3	[0.5–3.8]	0.566
>40	6	150	1					
Sex								
Male	13	207	1					
Female	15	165	1.45	[0.67–3.13]	0.344			
MS								
Married	8	214	1					
Unmarried	20	158	3.39	[1.46–7.89]	0.003	2.5	[0.9–6.6]	0.056
Education								
Yes	20	175	2.81	[1.21–6.54]	0.012	6.4	[0.8–48.1]	0.073
No	8	197	1					
PS								
Employed	13	235	1					
Unemployed	15	137	1.98	[0.91–4.28]	0.078	1.3	[0.6–2.9]	0.563
CP to HF								
Yes	14	183	1.03	[0.48–2.22]	0.934			
No	14	189	1					
CP to CP								
Yes	22	252	1.75	[0.69–4.43]	0.234	1.3	[0.5–3.4]	0.591
No	6	120	1					

SDC = Socio-demographic characteristics, GLN = Good level of knowledge, BA = Bivariate analysis, MA = Multivariate analysis, CP to HF = Close proximity to a health facility, CP to CP = Close proximity to a community pharmacy, MS = Marital status, PS = Professional status.

**Table 4:** Participants’ opinions towards antibiotic use and bacterial resistance, Rufisque, Senegal, 2017 (N = 400).

Statements	Yes, n (%)	No, n (%)	No Opinion, n (%)
Do you think that the population overuses antibiotics?	313 (78.3)	26 (6,5)	61 (15.3)
Do you think we give you enough information, during the consultations, about antibiotics regarding their ineffectiveness if they are misused?	112 (28)	278 (69.5)	10 (2.5)
Do you think we give you enough information, during the dispensation, about antibiotics regarding their ineffectiveness if they are misused?	214 (53.5)	181 (45.3)	5 (1.3)
Do you think you can play a big role in fighting bacterial resistance?	183 (45.8)	112 (28)	105 (26.3)
